# Public Baths

**Published:** 1895-10

**Authors:** 


### PUBLIC BATHS.

THE important part that cleanliness plays in the prevention of disease is recognised by every true physician. This is an inner truth that has only lately been recognised by statute. The last legislature of this state passed an act providing for the establishment of public baths and lavatories in all large cities, to be maintained in such numbers as the local board of health may determine to be requisite. This legislation was procured through the efforts of a sub-committee of the committee of seventy of New York city. We doubt if the importance of this law is appreciated by the public in general. The law makes it mandatory upon the board of health to determine as to the necessity of public bath houses, their location and number, as well as all essential details. The city authorities are then expected and required to furnish the necessary money to push forward the work to prompt and efficient completion. The responsibility of boards of health in this matter admittedly is very great, but they do not shrink from it.

In Buffalo, the board of health has already taken the proper initiative action to comply with this wholesome law, and the responsibility now lies with the common council to appropriate money with which to finish the good work. If this august body of statesmen should be slow, or niggardly, or captious in performing its part, the people will be pretty sure to call them to an account with a rapidity that may astonish them.

The idea of free public bath houses surely commends itself to all philanthropic and right-thinking people. The government has established them at Hot Springs, Ark., where their beneficial influences in the treatment of disease are recognised as well as their cleanly effects. All over the world they are exerting an influence for good. A clean man is not apt to be a bad man. Let Buffalo proceed to array herself in the front rank of this much needed reform.

